# Nanostripe-Confined Catalyst Formation for Uniform Growth of Ultrathin Silicon Nanowires

**DOI:** 10.3390/nano13010121

**Published:** 2022-12-26

**Authors:** Yinzi Cheng, Xin Gan, Zongguang Liu, Junzhuan Wang, Jun Xu, Kunji Chen, Linwei Yu

**Affiliations:** School of Electronic Science and Engineering/National Laboratory of Solid-State Microstructures, Nanjing University, Nanjing 210093, China

**Keywords:** silicon nanowires, confined catalyst formation, in-plane solid–liquid–solid growth

## Abstract

Uniform growth of ultrathin silicon nanowire (SiNW) channels is the key to accomplishing reliable integration of various SiNW-based electronics, but remains a formidable challenge for catalytic synthesis, largely due to the lack of uniform size control of the leading metallic droplets. In this work, we explored a nanostripe-confined approach to produce highly uniform indium (In) catalyst droplets that enabled the uniform growth of an orderly SiNW array via an in-plane solid–liquid–solid (IPSLS) guided growth directed by simple step edges. It was found that the size dispersion of the In droplets could be reduced substantially from $D_{\mathrm{cat}}^{pl}$ = 20 ± 96 nm on a planar surface to only $D_{\mathrm{cat}}^{ns}$ = 88 ± 13 nm when the width of the In nanostripe was narrowed to $W_{\mathrm{str}}=$ 100 nm, which could be qualitatively explained in a confined diffusion and nucleation model. The improved droplet uniformity was then translated into a more uniform growth of ultrathin SiNWs, with diameter of only $D_{\mathrm{nw}}=$ 28 ± 4 nm, which has not been reported for single-edge guided IPSLS growth. These results lay a solid basis for the construction of advanced SiNW-derived field-effect transistors, sensors and display applications.

## 1. Introduction

Silicon nanowires (SiNWs) are one of the promising 1D channel materials for the construction of high-performance field-effect transistors (FETs) [1,2,3], display logics [4,5,6] and sensors [7,8,9,10], due to their large surface-to-volume ratio and excellent electrostatic modulation capability in a fin or gate-all-around gating configuration. Compared to the sophisticated top-down patterning and etching strategy, the bottom-up catalytic growth of SiNWs, led by metal catalyst droplets, for example via the famous vapor–liquid–solid (VLS) growth mechanism [11,12,13,14,15], offers a low-cost, high-yield and diverse fabrication strategy. Many advanced nanoelectronics have been successfully demonstrated based on VLS-grown SiNWs serving as semiconducting channels [16,17,18,19]. However, a major challenge for the catalytic growth of SiNWs, in view of scalable electronic applications on planar substrate, is how to integrate or grow them directly into pre-designed locations without the use of post-growth transferring and alignment, while a uniform diameter control of the as-grown SiNWs, determined usually by the leading catalyst droplets [20,21], is also highly desirable.

**Figure 1:**
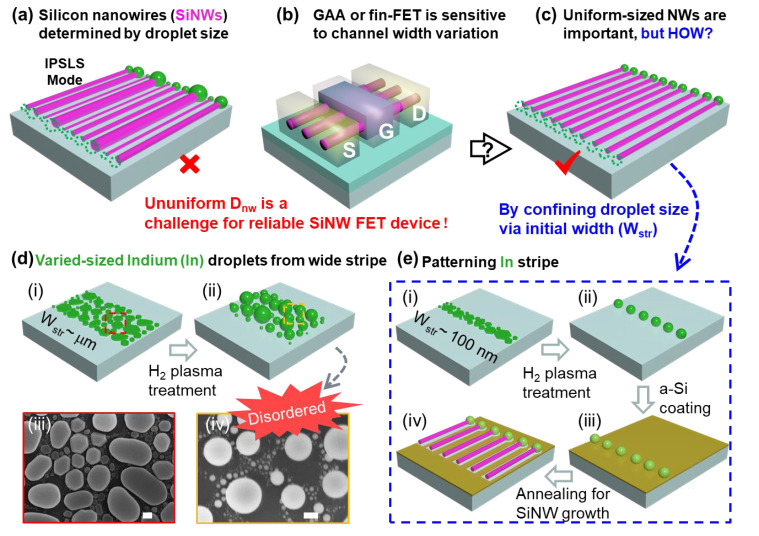
(**a**) Schematic illustration showing that the diameters of the SiNWs grown via IPSLS mode are determined by the size of the leading droplets. In view of serving as FET channels, in fin-gate or gate-all-around configurations, as depicted in (**b**), a uniform array of SiNW channels, for instance, those in (**c**), is highly desirable. (**d**) Panel (**i**,**ii**) diagram of the formation of random In catalyst droplets within the wide In stripes or planar surface, as observed in SEM images of the In grains before (**iii**) and after (**iv**) H_2_ plasma treatment. In comparison, (**e**) depicts the formation of rather uniform In droplets by using a narrow In stripe with a width down to 100 nm, with the key fabrication steps depicted in (**i**,**ii**) catalyst formation by H_2_ plasma treatment, (**iii**) amorphous silicon (a-Si) precursor coating, and (**iv**) annealing to activate the SiNW to grow by consuming the a-Si layer. Scale bars in panels (**iii**,**iv**) in (**d**) are all for 200 nm.

In order to gain better control of the catalytic growth of SiNWs, an in-plane solid–liquid–solid (IPSLS) growth strategy has been developed in our previous works [22,23,24], where a hydrogenated amorphous Si (a-Si) thin film is deposited upon a substrate surface to serve as the precursor layer for the indium (In) catalyst droplets to absorb and produce continuous polycrystalline SiNWs. In principle, the IPSLS growth of SiNWs is driven by the higher Gibbs energy in the disordered a-Si precursor layer, with respect to the crystalline phase of SiNWs, where the In droplets serve as a moving mediator to facilitate the phase conversion [22,23,25,26]. More importantly, the In catalyst droplets can be guided by pre-patterned edge lines to produce SiNW arrays along their moving courses [27,28,29], which is a key aspect that enables the scalable and precise integration of an orderly SiNW array. Similarly to that in VLS growth, the diameter of the IPSLS SiNWs is basically determined by the size of the leading catalyst droplets [22,30,31]. However, for the formation of the In catalyst droplets by using an H_2_ plasma treatment on a free planar surface or in relatively wide In stripes with a width >2 μm, as seen, for example, in Figure 1d(i,iii), the size dispersion of the In droplets is usually quite large (see, for instance, the SEM image in Figure 1d(iv)). Then, this random size variation in the catalyst droplets will be passed on to the as-grown SiNWs (Figure 1a), with larger diameter D_nw_ variation, which will pose a disadvantage to the device reliability [32], causing large fluctuations in the I_on/off_ ratio [33], mobility [34] and subthreshold swing (SS) [35,36], as diagrammed in Figure 1b. Therefore, it is of paramount importance to seek a new approach to greatly improve the diameter uniformity of the IPSLS SiNWs, which is indispensable for achieving a reliable electronic integration (Figure 1c). Actually, for the VLS growth of SiNWs, many catalyst formation control technologies have been explored to control the initial size of the catalyst metal droplets, such as via temperature control [37] and EBL pattern [38,39], which have proven rather efficient to produce highly uniform vertical VLS-grown SiNWs. Following these insights, the key to control the uniformity of IPSLS SiNWs is to accomplish, first and foremost, a uniform size control of the leading catalyst droplets, which unfortunately remains an unexplored topic for the IPSLS growth of SiNWs. In this work, a new nanostripe-confined catalyst formation approach is proposed and tested to obtain (Figure 1e), first, a uniform formation of In catalyst droplets, and then, use them to produce more uniform and ultrathin SiNWs with diameter of $D_{\mathrm{nw}}=28$ ± 4 nm, as a key basis for the future scalable and reliable integration of SiNW-based electronics.

## 2. Materials and Methods

### 2.1. Preparation of Catalyst Nanostripes

**Figure 2:**
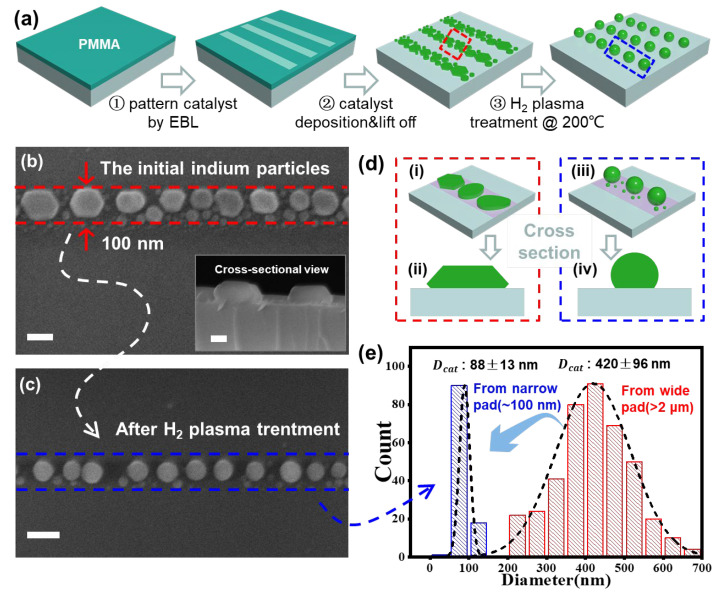
(**a**) Schematic illustration of the formation of uniform In droplets from narrow In stripes prepared by using EBL. (**b**) The SEM image of the initial discrete In catalyst grains evaporated on the nanostripe region with a width of 100 nm, while the inset shows the cross-sectional view of these pancake-shaped catalyst grains. (**c**) The formation of a chain of uniform In droplets after H_2_ plasma treatment. (**d**) Tilted and cross-sectional view diagrams of the In grains evaporated (**i**,**ii**) and after H_2_ plasma treatment (**iii**,**iv**) within the nanostripe regions. (**e**) Statistics of the droplet diameters formed within a strong nanostripe confinement (light blue, for narrow W_str_ = 100 nm) or on a much wider In stripe (light red, with W_str_ > 2 μm). Scale bars in (**b**,**c**) stand for 100 nm.

A silicon wafer coated with 500 nm thick SiO_2_ was first cleaned using acetone, alcohol and deionized water. The guiding edges, single-sided step edges, with etching depth of ~150 nm were prepared on the SiO_2_ surface by using standard photolithography and RIE etching procedures, as depicted in Figure 2a. Then, polymethyl methacrylate (PMMA) photoresist with thickness of 200 nm was spin-coated on the substrate at 3000 r/min. After that, a series of empty stripe regions with width (W_str_) ranging from 100 nm to 500 nm were patterned by electron beam lithography (EBL) in scanning electron microscopy with a dose value of 240. Finally, 16 nm In stripes were deposited via thermal evaporation, and the In stripes were obtained via standard lift-off procedure.

### 2.2. Growth of Ultrathin SiNW Array

The samples were loaded into a PECVD system and treated by H_2_ plasma under 200 °C, with H_2_ flow of 14 SCCM and pressure of 140 Pa. An H_2_ plasma treatment was performed to produce H^+^ radicals and reduce the thin In_2_O_3_ oxide layer formed on the surface of the indium grains, so as to release them to deform and merge into discrete and spherical droplets. Then, the 10 nm thick a-Si:H precursor layer was deposited at 100 °C, with a gas flow rate and chamber pressure of 5 SCCM and 20 Pa, respectively. After that, the substrate temperature was raised to ~350 °C and kept in vacuum for 1 h. The extra a-Si supply on the vertical sidewall surfaces attracted the In droplets to move along the edge lines to produce SiNW arrays by converting the a-Si layer into crystalline SiNWs. Finally, the remnant a-Si layer was selectively etched off by using H_2_ plasma at ~120 °C for 5 min, with typical gas flow rate and chamber pressure of 15 SCCM and 140 Pa, respectively.

## 3. Results and Discussion

### 3.1. Formation of Uniform In Droplets from Narrow In Stripes

After H_2_ plasma treatment, the surface oxide layers of the In catalysts were removed, and the initial faceted and irregular In grains (see the SEM characterization in Figure 2b, for example) were transformed into discrete spherical droplets (Figure 2c). Actually, for the In stripe of W_str_ = 100 nm, the initial In grains were of flat pancake shapes, as witnessed in the cross-sectional SEM view provided in the inset of Figure 2b and illustrated in Figure 2d(i,ii), with many random small grains resting among the larger ones. After H_2_ plasma treatment at a temperature higher than the In melting point of 156 °C, the In droplets were allowed to transform into energetically more favorable spherical shapes, as observed in Figure 2c and depicted in Figure 2d(iii,iv), while the closely neighbored In grains could also agglomerate into bigger ones. Interestingly, compared to the size distribution of the In droplets formed on the planar surface (or within much wider stripes $W_{\mathrm{str}}>2\;\mu m$, the red columns in Figure 2e), the size dispersion of the In droplets within narrow stripes (blue, for W_str_ = 100 nm) could be substantially reduced from $D_{\mathrm{In}}^{2\mu m}=$20 ± 96 nm to $D_{\mathrm{In}}^{100nm}=88$ ± 13 nm, which is highly beneficial for achieving uniform size control of the In catalyst droplets and thus the as-grown SiNWs.

### 3.2. In Droplet Formation on Nanostripes of Different Widths

**Figure 3:**
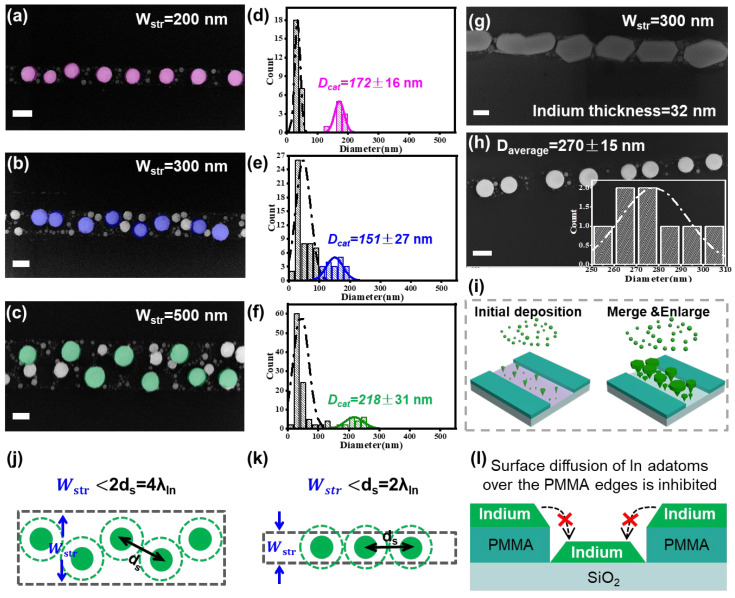
(**a**–**c**) SEM images of the In droplets formed on the nanostripes with the same In thickness of 16 nm but different W_str_ = 200 nm, 300 nm and 500 nm, with corresponding diameter statistics displayed in (**d**–**f**). In comparison, the In catalyst formations within a stripe width of 300 nm, with a thicker In thickness of 32 nm, are presented in (**g**,**h**) for the situations of initial grains and the droplets formed after H_2_ plasma treatment. (**i**) depicts schematically the (left) initial In atom nucleation and (right) subsequent merging stages during the thermal evaporation. (**j**,**k**) illustrate the formation of discrete In nucleation sites and their collection zones, delineated by the dashed circles, within nanostripe widths of W_str_ ~ 3$\lambda_{In}$ and W_str_ ~ 2$\lambda_{In}$, respectively. (**l**) illustrates that the surface diffusion of In adatoms over the PMMA pattern edges is very inefficient or inhibited. Scale bars in (**a**–**c**,**g**,**h**) are all for 200 nm.

In order to understand how the nanostripe confinement helped to improve the uniformity of the catalyst formation, a series of In stripes with different widths of 200 nm, 300 nm and 500 nm were prepared by using EBL, with a constant In thickness of 16 nm. Indeed, as seen in Figure 3a–c and the corresponding statistics in Figure 3d–f, there was a clear trend where the size dispersion of the relatively large In droplets, highlighted by different colors, could be gradually improved from $D_{\mathrm{In}}^{500nm}=$ 218 ± 31 nm to $D_{\mathrm{In}}^{300nm}=$ 151 ± 27 nm and $D_{\mathrm{In}}^{200nm}=$ 172 ± 16 nm, with the decrease in the In stripe width. Meanwhile, there was also a high-density population of much smaller droplets, with typical diameter of ~50 nm, among the larger ones, which all had a similar size distribution for different In stripe widths. In addition, for the same stripe width of 300 nm (Figure 3b), if the In thickness was increased from 16 nm to 32 nm, the In grains were found to merge with their neighbors, as seen in Figure 3g, while the resulting In droplets after H_2_ plasma treatment became not only larger in diameter but also far more uniform than those observed in Figure 3b, with $D_{\mathrm{In}\_32\mathrm{nm}}^{300nm}=$ 270 ± 15 nm (Figure 3h).

These catalyst droplet formation phenomena could be explained based on the following considerations: at the very beginning of the evaporation, the In atoms, evaporated by thermal evaporation, will land on the sample surface and diffuse on the empty surface until they run into each other to form more stable nuclei (as depicted schematically by the left panel of Figure 3i). In the next step, more adatoms continue to arrive and get trapped by the nearest nuclei (the right panel of Figure 3i) if they fall within the corresponding surface collection zone, which is roughly measured by the typical diffusion length of the In atoms $\lambda_{In}$. On a planar surface, the area of the collection zone can be approximated by $S_{ad\_planar}\sim d_{s}^{2}\sim 4\lambda_{In}^{2}$, where $d_{s}$ is the separation between the initial nucleation sites or grains $d_{s}\sim 2\lambda_{In}$. According to the average large grain-to-grain separations, extracted from the droplets on the planar surface, as seen, for example, in Figure S1, the average separation is estimated from the density by $d_{s}=n_{In}^{-1/2}\sim 244\;nm$, or a $\lambda_{In}\sim 122\;nm$.

Moreover, for the nanostripe-confined catalyst formation, the surface diffusion of the In adatoms on the PMMA resistor polymer surface is considered to be very inefficient, and thus, there is likely little flux contribution coming from the PMMA surface to the stripe regions, as schematically depicted in Figure 3l. So, for the initial nucleation formation within a nanostripe region with $W_{str}<d_{s}\;or\;2\lambda_{In}$, the effective area of the adatom collection zone is reduced and becomes $S_{ad\_str}=d_{s}\;W_{str}<d_{s}^{2}$.

In this scenario, the volume of the final catalyst droplets formed within a nanostripe or on a planar surface can be written as $D_{In\_str}^{3}\sim S_{ad}\;t_{In}=d_{s}\;W_{str}\;t_{In}$ and $D_{In\_planar}^{3}\sim S_{ad}\;t_{In}=d_{s}^{2}\;t_{In}$, respectively. Considering the random variation of the grain-to-grain separation $\delta d_{s},$ that is $d_{s}=\bar{d_{s}}+\delta d_{s}$, as the major source of size fluctuations, its influence on the final diameter dispersions/variations of the In droplets within a nanostripe or on a planar surface can be derived as,

(1) $$\delta D_{In\_str}\sim \delta d_{s}\frac{W_{str}\;}{3D_{In}^{2}/t_{In}}\;$$

(2) $$\delta D_{In\_planar}\sim \delta d_{s}\frac{2d_{s}}{3D_{In}^{2}/t_{In}}\;$$

Obviously, for a narrow In stripe confinement, $W_{str}\ll d_{s}$, the random diameter fluctuation passed to the catalyst droplets can be greatly suppressed within the nanostripe, as $\delta D_{In\_str}\ll \delta D_{In\_planar}$. Additionally, it can be seen from both Equations (1) and (2) that, with a larger In catalyst droplet, the diameter fluctuation should also decrease, because of the denominator term of $3D_{In}^{2}/t_{In}$.

Meanwhile, the formation of the tiny grains among the large ones should happen at a later stage, that is, after the formation of the large grains. This can be clearly seen from the cross-sectional SEM view in Figure S2, where the small grains only exist in the spare regions among the large grains, with a much lower height than their larger neighbors. The emergence of such small grains seems to indicate that, with the increase in more In coverage on the sample surface, the irradiation heating (from the evaporation crucible below) on the sample is gradually decreased, probably due to the enhanced reflection of In layer coating deposited on the sample surface. This thus leads to a temperature decrease on the sample surface that quickly reduces the diffusion distance of the adatoms on the surface, as $\lambda_{In}\sim \lambda_{0}e^{-E_{d}/kT}$ is highly temperature-dependent, where $E_{d}$ is the diffusion barrier height on the SiO_2_ substrate surface. So, confining the catalyst formation within a narrow stripe region, with $W_{str}\ll 2\;d_{s}$ at least during the initial nucleation stage, provides indeed a convenient and efficient way to suppress the random size fluctuation of the catalyst droplets (seen in Figure 3j,k). It is also predicted that maintaining suitable heating on the substrate holder, though not available for the current experimental setup in this work, could help to further improve the uniformity of the In catalyst droplets.

### 3.3. Growth of Ultrathin SiNWs Led by the Uniform In Droplets

**Figure 4:**
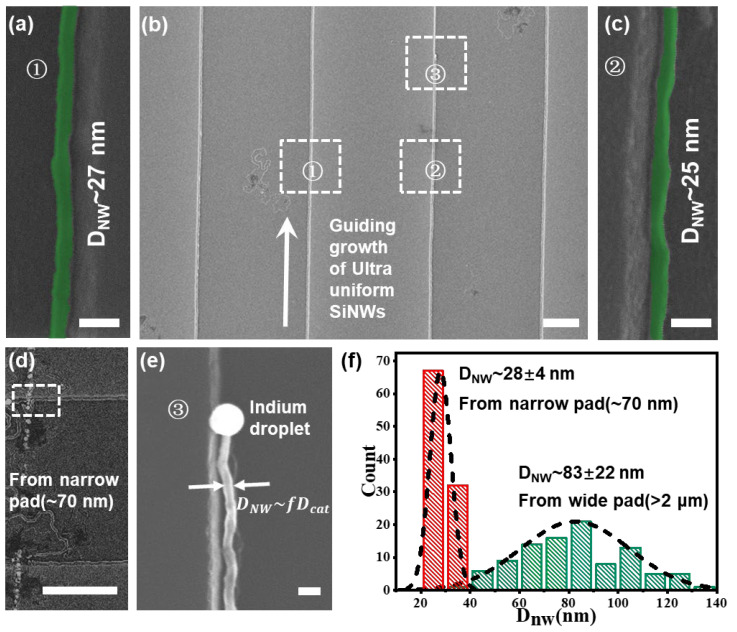
(**a**–**c**) SEM images of the IPSLS growth of uniform ultrathin SiNWs led by the uniform In droplets formed within nanostripes of W_str_ ~ 70 nm, with diameters of 27 nm and 25 nm in (**a**) and (**c**), respectively. Close view of the (**d**) starting and (**e**) ending of a specific SiNW guided along the step edge. (**f**) The diameter statistics of the SiNWs grown from narrow In stripe (red) and wide stripe (green). Scale bars in (**a**,**c**,**e**) are for 100 nm, (**b**,**d**) stand for 1 μm.

Since the SiNW diameter was basically determined by the leading In catalyst droplets via IPSLS strategy, with D_nw_ ~ D_In_ [22,30,31] (for instance, that in Figure 4e), ultrathin SiNW arrays, as seen in Figure 4a–c, could now be grown by using the pre-patterned In nanostripes with W_str_ = 70 nm; see Figure 4d for more information on the starting growth location (and Figure S3 for the initial catalyst formation prior to annealing growth). Remarkably, rather thin and uniform SiNWs with an average diameter of D_nw_ = 28 ± 4 nm, according to the statistics in Figure 4f, could be grown directly by using only single-step guided IPSLS growth, which is far more uniform and thinner, compared to the SiNWs grown by using catalysts on much wider micro stripes (W_str_ > 2 μm), as seen also with the corresponding droplets in Figure S4 with an average diameter of D_nw_ = 83 ± 22 nm. The diameter fluctuation along the SiNWs was caused mostly by the roughness of the guiding edge. In addition, the catalyst droplets will remain at the end of SiNWs after growth by using the IPSLS strategy (Figure 4e) and can be easily removed by using dilute hydrochloric acid before the fabrication of high-performance devices. These results indicate a new narrow-stripe-confined catalyst formation strategy to obtain uniform indium catalyst droplets, as a key to substantially suppress the SiNW-to-SiNW diameter variation. As a matter of fact, the construction of high-performance SiNW-based logics and sensors [31,40] all demand rather thin, uniform and orderly SiNW arrays as 1D channels to achieve stronger electrostatic modulation control [41] or higher field-effect sensitivity [42].

## 4. Conclusions

In summary, we have established a nanostripe-confined approach for rather uniform In catalyst formation and demonstrated an orderly growth of ultrathin SiNW arrays, with a narrow size dispersion of only D_nw_ = 28 ± 4 nm, enabled by the largely improved catalyst droplet diameter uniformity in pre-patterned nanostripe regions, which can help to largely suppress the random diffusion and nucleation of the In catalyst adatoms particularly during the early grain formation stage within the tightly confined nanostripe. This study opens up a reliable route to batch fabricate and integrate ultrathin SiNW channels for various high-performance electronics and sensor applications.

## Data Availability

Data are contained within the article and Supplementary Materials.

## Supplementary Materials

The following supporting information can be downloaded at: https://www.mdpi.com/article/10.3390/nano13010121/s1, Figure S1: SEM image of the catalyst droplet dispersion on planar surface after H_2_ plasma treatment. The average separation can be estimated from the density by $d_{s}=n_{In}^{-1/2}\sim 244\;nm$; Figure S2: (a) SEM image of In grains deposited on planar surface. (b) Cross-sectional view of the deposited In grains. The small grains only exist in the spare regions among the large grains, with a much lower height than their larger neighbors; Figure S3: (a) SEM image of In droplets formed on 70 nm stripe after H_2_ plasma treatment. (b) Diameter statistics of the as-received In droplets, with D_cat_ of 55 nm ± 11 nm; Figure S4: Guided growth of SiNW arrays via the IPSLS mode by using catalysts formed within wide stripe (>2 μm), which shows the large diameter variation of formed catalysts (a) and the as-grown SiNW (b).

## Abbreviations

SiNW: Silicon nanowire; NW: Nanowire; GAA: Gate-All-Around; cat: Catalyst; str: Stripe; VLS: Vapor-Liquid-Solid; FETs: Field effect transistors; IPSLS: In-plane solid-liquid-solid; SS: Subthreshold swing; EBL: Electron beam lithography; PMMA: Polymethyl methacrylate; RIE: Reactive ion etching; a-Si:H: Hydrogenated amorphous silicon; a-Si: Amorphous silicon; In: Indium; W_str_: Width of stripe; D_nw_: Diameter of the nanowire; D_In_: Diameter of the indium; **λ**_In_: The diffusion length of indium; d_s_: The separation between the nucleation.
