# Supplementary material for: Nanostripe-Confined Catalyst Formation for Uniform Growth of Ultrathin Silicon Nanowires
Source: Nanomaterials (Basel). 2022 Dec 26;13(1):121. doi: 10.3390/nano13010121 (PMC9824257; doi:10.3390/nano13010121)
Supplement: Supplementary file 1 [file nanomaterials-13-00121-s001.zip › nanomaterials-2071200-supplementary.pdf]

Supplementary Materials for

Nanostripe-confined catalyst formation for uniform growth of ultrathin silicon nanowires

Yinzi Cheng[1], Xin Gan[1], Zongguang Liu[1, *], Junzhuan Wang[1], Jun Xu[1], Kunji Chen[1] and Linwei Yu[1, *]

[1] School of Electronic Science and Engineering/National Laboratory of Solid-State Microstructures, Nanjing University, 210093 Nanjing, China

**Corresponding Email: yulinwei@nju.edu.cn; liuzongguang@nju.edu.cn*

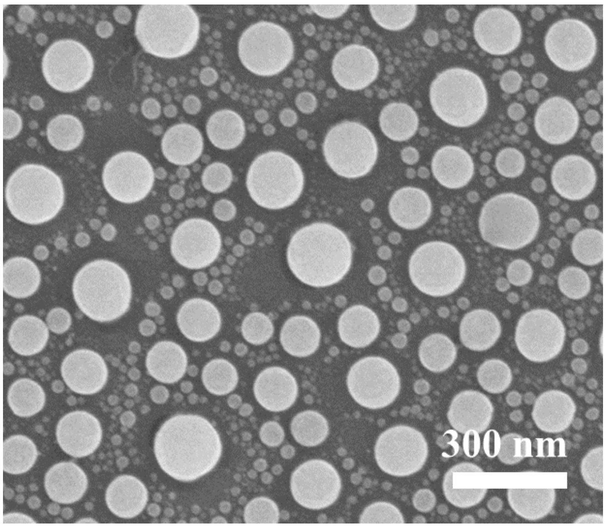

Figure S1. The SEM image of the catalyst droplet dispersion on planar surface after H_2 plasma treatment. The average separation can be estimated from the density by $d_s = n_{In}^{-1/2} \sim 244\ nm$.

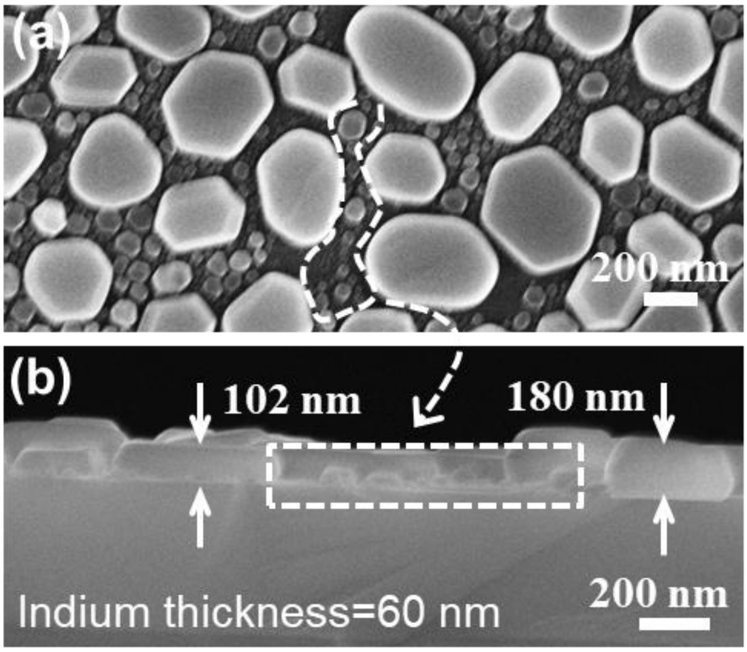

Figure S2. (a) The SEM image of In grains deposited on planar surface. (b) The cross-section view of the deposited In grains. The small grains only exist in the spare regions among the large grains, with a much lower height than their larger neighbors.

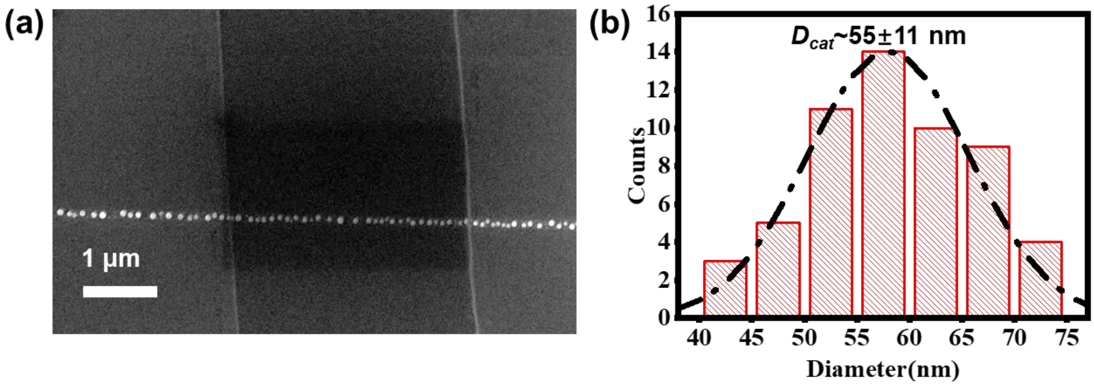

Figure S3. (a) SEM image of In droplets formed on 70 nm stripe after H_2 plasma treatment. (b) The diameter statistics of the as-received In droplets, with D_{cat} of 55 nm±11 nm.

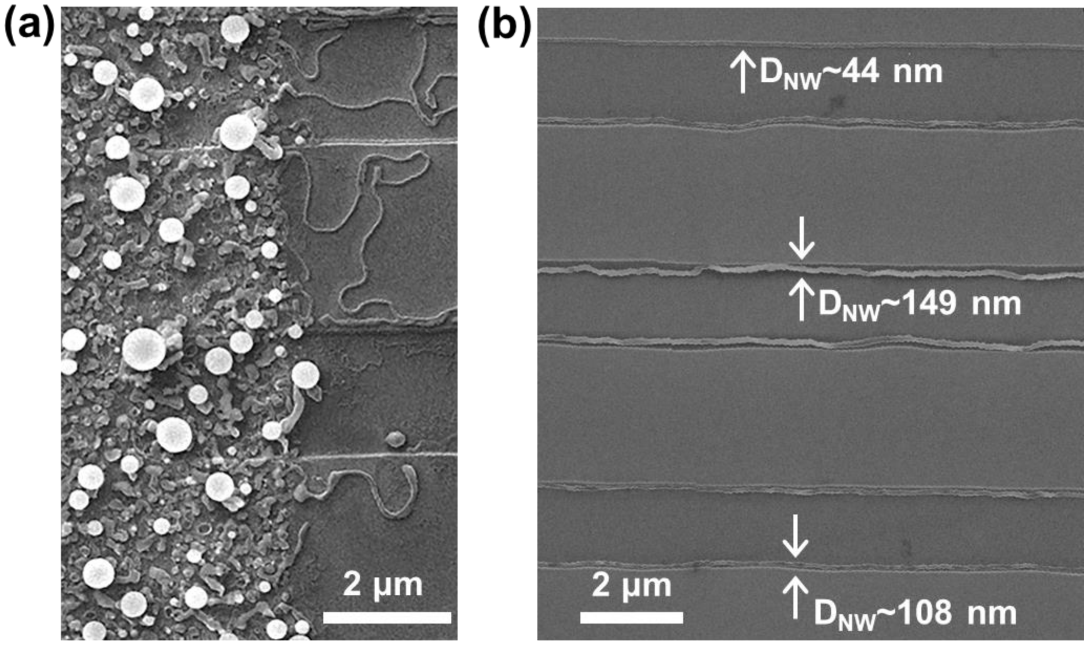

Figure S4. The guided growth of SiNW arrays via the IPSLS mode by using catalysts formed within wide stripe (> 2μm), which shows the large diameter variation of formed catalysts (a) and the as-grown SiNW (b).
